# Therapeutic efficiency of human amniotic epithelial stem cell-derived functional hepatocyte-like cells in mice with acute hepatic failure

**DOI:** 10.1186/s13287-018-1063-2

**Published:** 2018-11-21

**Authors:** Quan-Wen Liu, Qian-Yu Liu, Jing-Yuan Li, Li Wei, Kang-Kang Ren, Xiang-Cheng Zhang, Ting Ding, Ling Xiao, Wen-Jie Zhang, Han-You Wu, Hong-Bo Xin

**Affiliations:** 1 Institute of Translational Medicine, Nanchang University, No. 1299 Xuefu Road, Honggutan District, Nanchang, 330031 Jiangxi Province People’s Republic of China; 2 Department of Obstetrics and Gynecology, The First Affiliated Hospital of Nanchang University, 17 Yongwaizheng Road, Nanchang, 330006 Jiangxi Province People’s Republic of China; 3 School of Life and Science, Nanchang University, No. 1299 Xuefu Road, Honggutan District, Nanchang, 330031 Jiangxi Province People’s Republic of China

**Keywords:** Acute liver failure, Human amniotic epithelial stem cells, Hepatocyte-like cells, Human primary hepatocytes, Cell transplantation

## Abstract

**Background:**

Hepatocyte transplantation has been proposed as an effective treatment for patients with acute liver failure (ALF), but its application is limited by a severe shortage of donor livers. Human pluripotent stem cells (hPSCs) have emerged as a potential cell source for regenerative medicine. Human amniotic epithelial stem cells (hAESCs) derived from amniotic membrane have multilineage differentiation potential which makes them suitable for possible application in hepatocyte regeneration and ALF treatment.

**Methods:**

The pluripotent characteristics, immunogenicity, and tumorigenicity of hAESCs were studied by various methods. hAESCs were differentiated to hepatocyte-like cells (HLCs) using a non-transgenic and three-step induction protocol. ALB secretion, urea production, periodic acid-Schiff staining, and ICG uptake were performed to investigate the function of HLCs. The HLCs were transplanted into ALF NOD-SCID (nonobese diabetic severe combined immunodeficient) mouse, and the therapeutic effects were determined via liver function test, histopathology, and survival rate analysis. The ability of HLCs to engraft the damaged liver was evaluated by detecting the presence of GFP-positive cells.

**Results:**

hAESCs expressed various markers of embryonic stem cells, epithelial stem cells, and mesenchymal stem cells and have low immunogenicity and no tumorigenicity. hAESC-derived hepatocytes possess the similar functions of human primary hepatocytes (hPH) such as producing urea, secreting ALB, uptaking ICG, storing glycogen, and expressing CYP enzymes. HLC transplantation via the tail vein could engraft in live parenchymal, improve the liver function, and protect hepatic injury from CCl_4_-induced ALF in mice. More importantly, HLC transplantation was able to significantly prolong the survival of ALF mouse.

**Conclusion:**

We have established a rapid and efficient differentiation protocol that is able to successfully generate ample functional HLCs from hAESCs, in which the liver injuries and death rate of CCl_4_-induced ALF mouse can be significantly rescued by HLC transplantation. Therefore, our results may offer a superior approach for treating ALF.

## Background

Acute liver failure (ALF), a lethal syndrome, is characterized by acute deterioration of liver function, widespread hepatocyte necrosis, and subsequent multiorgan failure [1], and the mortality rate is as high as 90% [2, 3]. Currently, orthotopic liver transplantation (OLT) followed by life-long administration of immunosuppressant is the most common and effective therapy for treatment of ALF [4, 5]; however, its application is limited by a severe shortage of donor organs and high cost [6]. Hepatocyte transplantation has been proposed as an alternative strategy instead of whole-organ transplantation for treatment of ALF [7]. Unfortunately, the lack of donor livers also makes it difficult to obtain sufficient numbers of functional hepatocytes [8]. To solve this dilemma, novel strategies for generating ample functional hepatocytes are greatly needed [9].

Human pluripotent stem cells (hPSCs) have the capability of self-renewal and differentiating into derivates of the three embryonic layers, which make them suitable candidates for possible application in hepatocyte regeneration and ALF treatment [10, 11]. It has been proposed that hepatocytes derived from human embryonic stem cells [12, 13], induced pluripotent stem cells [14], and mesenchymal stem cells [8, 15–17] can serve as a potential source for acute liver failure therapy. However, there are still many questions remain unanswered about the use of stem cells to treat ALF [18], including tumorigenicity, immune incompatibility between donor and receptor, and ethical issues [19, 20]. To overcome these shortfalls, the generation of functional hepatocyte from new source of stem cells is necessary. Recently, human amniotic epithelial stem cells (hAESCs) have been recognized as one of the most promising stem cells [21–24]. It has been reported that the stem cells derived from term amnion possess multilineage differentiation potential [25], non-tumorigenicity [26], low immunogenic profile [27], and anti-inflammatory functions [28], reducing the risk of cell rejection and the potential of graft-versus-host disease. In addition, since hAESCs have similar pluripotent properties with embryonic stem cells, they are not associated with the ethical concerns [29]. Taken together, these observations suggest that the hAESCs might be ideal candidates for regenerative medicine and tissue repair.

Recently, we isolated hAESCs from human amniotic membrane and characterized their morphology, phenotypic profiles, and growth potency. Here, we described an efficient three-step differentiation protocol for generation of hepatocyte-like cells (HLCs) from hAESCs in vitro by using selective cell factors and small molecule compounds. The HLCs could express hepatic-specific genes and possess the functional properties of mature hepatocytes, including secretion of albumin (ALB), production of urea, uptake of indocyanine green, and stockpile of glycogen. Finally, the therapeutic effects of HLCs were evaluated following transplantation into a carbon tetrachloride (CCl_4_)-induced nonobese diabetic severe combined immunodeficient (NOD-SCID) mouse model of acute hepatic failure in vivo. We demonstrated that the HLCs could improve the liver function, protect hepatic injury, and rescue the acute hepatic failure mice. These results suggest that the HLCs derived from hAESCs may serve as a novel therapy for ALF patients in the clinic.

## Methods

### Isolation, culture, and expansion of hAESCs

Human fetal placentas were obtained from the Department of Obstetrics and Gynecology, The First Affiliated Hospital of Nanchang University. The verbal consent was obtained from all of the volunteers prior to their participation. The research procedure was approved by the ethics committee of The First Affiliated Hospital of Nanchang University. hAESCs were prepared as previously described [23]. Fetal placentae were sampled and transported to the laboratory in sterile Hanks’ balanced salt solution (HBSS; calcium- and magnesium-free). The amniotic membrane was mechanically peeled from the underlying chorion layer of the placenta body and extensively washed two to three times with HBSS to remove blood. Next, the amniotic membranes were treated with 0.25% trypsin-EDTA (Thermo Fisher, Nanchang, China) for 60 min in a humidified incubator with 5% CO_2_ at 37 °C. Single-cell suspension was obtained after filtering the tissues through a 70-μm cell strainer (BD Labware, Shanghai, China), and the digestion was terminated by addition of medium containing 10% FBS. The single-cell suspension was centrifuged at 1000 rpm for 5 min. The supernatant was discarded, and the cells were re-suspended by DMEM-high glucose supplemented with 10% FBS, 2 mM L-glutamine, 1% antibiotic-antimycotic, 1% sodium pyruvate, 1% nonessential amino acids, 55 μM β-mercaptoethanol, 100 U/ml penicillin and streptomycin (all from Thermo Fisher), and 10 ng/ml epidermal growth factor (EGF; Peprotech, Rocky Hill, NJ, USA). hAESCs were placed in cell culture dishes (Corning, NY, USA) at a density of 5 × 10^4^ cells/cm^2^ at 37 °C in a 5% CO_2_ incubator. Unattached cells and debris were removed after 3 days. In each experiment, the cells were grown to approximately 80% confluence, and only cells between passages 2 and 5 were used for subsequent experiments.

### Reverse transcription-polymerase chain reaction (RT-PCR)

Total RNA from each sample was extracted by the Trizol reagent (Thermo Fisher). Purity was assessed by the absorbance ratio at 260 and 280 nm. RNA (100 ng to 1 μg) was reverse transcribed into cDNA with the M-MLV Reverse Transcriptase (Promega, Shanghai, China) according to the manufacturer’s instructions. The primers for the target products were designed as in Table 1. Polymerase chain reactions (PCR) were carried out in a PCR thermal cycler (Thermo Hybaid, Waltham, MA, USA). PCR products were electrophoresed on a 1.0% (*m*/*v*) agarose gel containing 0.5 μg/ml ethidium bromide for nucleic acid visualization under UV light. In parallel, mRNA levels of human housekeeping GAPDH were analyzed as an internal normalization control.

**Table 1:** Primers and conditions used for RT-PCR to detect gene transcripts in hAESCs, HPCs, HLCs, and hPH

Genes	Sequence	TM(°C)	Size(bp)
GAPDH	F:5′-CCACCCATGGCAAATTCCATGGCA-3′	59	598
	R:5′-TCTAGACGGCAGGTCAGGTCCACC-3′		
Nanog	F:5′-CAATGGTGTGACGCAGGGAT-3′	52	149
	R:5′-TGCACCAGGTCTGAGTGTTC-3′		
OCT4	F:5′-ATCCCTGAACCTAGTGGGGA-3′	59	480
	R:5′-CACTCGGACCACATCCTTCT-3′		
E-cadherin	F:5′-GGAAAATGCCCAGCCCGAC-3′	60	210
	R:5′-ACGTTATATGCCCGCACGAT-3′		
CK7	F:5′-TTTGTGGTGCTGAAGAAGGATG-3′	57	683
	R:5′-CCACCAGTGGAATTCATCACA-3′		
CD105	F:5′-TCCTCCCAAGGACACTTGTA-3′	57	244
	R:5′-CGCCTCATTGCTGATCATAC-3′		
CD73	F:5′-CTCCTCTCAATCATGCCGCT-3′	55	174
	R:5′-TGGATTCCATTGTTGCGTTCA-3′		
CD90	F:5′-GAGGGAGGAAGAGCAGACCT-3′	57	896
	R:5′-CCTGGATCGGGTTATGATGGG-3′		
HLA-DR	F:5′-CAAGAGCTCCAACTATACTCCGAT-3′	63	273
	R:5′-ACCCTGCAGTCGTAAACGTCC-3′		
HLA-ABC	F:5′-GTATTTCTTCACATCCGTGTCCCG-3′	65	394
	R:5′-GTCCGCCGCGGTCCAAGAGCGCAG-3′		
ALB	F:5′-GGTGAGACCAGAGGTTGATGTG-3′	61	695
	R:5′-GCAGCAGCACGACAGAGTAATC-3′		
AAT	F:5′-TGAGTTCGCCTTCAGCCTATACC-3′	61	530
	R:5′-AGTCCTCTTCCTCGGTGTCCTT-3′		
CYP3A4	F:5′-ATGGTCAACAGCCTGTGCT-3′	57	317
	R:5′-CATGCTGTAGGCCCCAAAGA-3′		
CK19	F:5′-GCTTTGTGTCCTCGTCCTCC-3′	57	614
	R:5′-TTGGCTTCGCATGTCACTCA-3′		
CK18	F:5′-ATGAGCTTCACCACTCGCTC-3′	57	688
	R:5′-TGGCAATCTGGGCTTGTAGG-3′		
CK7	F:5′-CCCAGACATCTTTGAGGCCC-3′	57	973
	R:5′-TTCACGGCTCCCACTCCATC-3′		
AFP	F:5′-AGTTTAGCTGACCTGGCTACC-3′	55	275
	F:5′-GGAGTGGGCTTTTTGTGTGC-3′		

*F* forward primer, *R* reverse primer

### Flow cytometry analysis

The cultured hAESCs were characterized by flow cytometry. Cells were washed and resuspended at a concentration of 1 × 10^6^ cells/ml in staining buffer (PBS). Cells were incubated in the dark at 2–8 °C with antibodies against mesenchymal stem cell markers (CD29-FITC, CD73-PE, CD90-FITC, and CD105-PE), hematopoietic cell markers (CD34-PE and CD45-FITC), and major histocompatibility (HLA-ABC-PE and HLA-DR-FITC) (all from BD Biosciences). After 30 min, the cell suspensions were washed twice and resuspended in 200 μl PBS for flow cytometry (FACS Aria, BD Biosciences) using FLOWJO TM software (TreeStar, Inc., Ashland, OR, USA).

### Immunofluorescence

For immunolabeling, cells were fixed in prechilled PBS with 4% paraformaldehyde for 15 min and permeabilized in PBS with 0.25% Triton X-100 for 10 min at room temperature. Nonspecific binding sites were blocked for 1 h by PBS containing 1% bovine serum albumin and 0.1% Tween 20. The fixed cells were incubated overnight at 4 °C with antibodies specific for OCT4 (5 μg/ml, rabbit polyclonal, Abcam, Nanchang, China), SSEA-4 (15 μg/ml, mouse monoclonal, Abcam), Nanog (1:200, rabbit monoclonal, Abcam), E-cadherin (1:100, mouse monoclonal, Abcam), Sox17 (1:50, mouse monoclonal, Abcam), FOXA2 (1:350, rabbit monoclonal, Abcam), AFP (5 μg/ml, mouse monoclonal, Abcam), ALB (1:500, rabbit monoclonal, Abcam), and AAT (1:50, rabbit monoclonal, Abcam). Specific labeling was visualized using secondary donkey anti-mouse or anti-rabbit antibodies conjugated to either Alexa Fluor 488 or Alexa Fluor 568 (Jackson, Nanchang, China). Nuclei were visualized by staining with DAPI (Thermo Fisher).

### Animal models

NOD-SCID male mice at age of 8-week-old were purchased from Changsha SLAC Laboratory Animal Company (Changsha, China, http://www.hnsja.com/) and maintained on 12-h light/dark cycles with food and water available ad libitum at the Laboratory Animal Center of Institute of Translational Medicine of Nanchang University. All animal procedures described here were reviewed and approved by the Animal Care and Use Committee of Nanchang University.

### Soft agar tumorigenicity test

The bottom layer of soft agar (0.6%) was prepared into 6-well plates, and hAESCs were plated onto the upper layer of soft agar (0.3%) at 1 × 10^3^/well and incubated at 37 °C with 5% CO_2_ for 30 days. Human liver carcinoma cell HepG2 was used as the control. The colonies were observed and imaged by phase contrast microscopy.

### In vivo tumorigenicity test

hAESCs were suspended at 2.5 × 10^7^ cells/ml in PBS. NOD-SCID mice were anesthetized with pentobarbital. We injected 200 μl of the cell suspension (5 × 10^6^ cells) into the right back and left thigh muscle of NOD-SCID mice, respectively. The same number of embryonic stem cells was used as positive controls. We observed the tumor forming every day for up to 20 weeks.

### Differentiation of hAESCs into HLCs in vitro

hAESCs between passage 2 and 5 were planted on the Matrigel (Thermo Fisher)-coated dishes at a density of 5 × 10^4^ cells/cm^2^ and cultured in expansion medium at 37 °C with 5% CO_2_. Hepatogenic induction was conducted as follows. Once the cells reached to 90% confluence, the medium was changed to IMDM (Thermo Fisher) containing 10% FBS, 100 mM nonessential amino acid, 2 mM L-glutamine, 55 μM β-mercaptoethanol, 100 U/ml penicillin/streptomycin, 10 ng/ml EGF (Peprotech, Nanchang, China), and 10 ng/ml bFGF (Peprotech) for 2 days. During the next step, the medium was replaced with IMDM supplemented with 10% FBS, 100 mM nonessential amino acid, 2 mM L-glutamine, 55 μM β-mercaptoethanol, 100 U/ml penicillin/streptomycin, 10 ng/ml EGF, 10 ng/ml bFGF, 20 ng/ml HGF (Peprotech), 1 μM dexamethasone (Thermo Fisher), and 1% (*v*/*v*) ITS Premix (Thermo Fisher) for 5 days. Finally, the cells were treated with IMDM supplemented with 10% FBS, 100 mM nonessential amino acid, 2 mM L-glutamine, 55 μM β-mercaptoethanol, 100 U/ml penicillin/streptomycin, 10 ng/ml EGF, 20 ng/ml oncostatin M (OSM; St Louis, MO, USA), 20 ng/ml HGF, 1 μM dexamethasone, and 1% (*v*/*v*) ITS Premix for 7 days. The differentiation medium was changed every 2 days.

### Isolation of human primary hepatocytes (hPH)

Liver tissue resection surplus was collected on ice and transported to the laboratory for processing as soon as possible. The tissue was cut into small pieces and washed in HBSS to remove excess blood under aseptic conditions. The specimens were treated with pre-warmed EGTA buffer (HBSS, 0.5 mM EGTA, 0.5% bovine serum albumin) and incubated at 37 °C for 10 min with gentle shaking at 120 rpm in a water bath. Tissue was then washed three times in HBSS to remove remaining blood and EGTA and digested with pre-warmed digestion buffer (HBSS, 0.05% collagenase IV, 0.5% BSA, 10 mM CaCl_2_) and agitated (100 rpm) in a water bath with shaking bed for 30 min at 37 °C. Following the digestion procedure, the stop solution (DMEM, 10% FBS) was immediately added into the digestion buffer for enzyme inactivation to prevent the over-digestion of the tissue. The cell solution was then filtered through a 70-μm cell strainer, centrifuged (80*g* for 5 min at 4 °C), and washed with ice-cold PBS. The cell solution was centrifuged again, and the pellet was re-suspended in hepatocyte incubation medium (WME glutamax supplemented with 10% FBS, 32 mU/ml human insulin, 1 mM sodium pyruvate, 1% MEM, 15 mM HEPES, 0.8 mg/ml hydrocortisone, and 100 U/100 mM penicillin/streptomycin). Hepatocytes were seeded on six-well plates that were previously coated with rat tail collagen (1 mg/ml, Roche) at a density of 1.5 × 10^5^ cells/cm^2^. The cells were cultured in a humidified incubator at 37 °C, 5% CO_2_ for at least 4 h. After 4 h, the hepatocytes have been adhered and the medium was replaced with fresh hepatocyte incubation medium.

### Real-time quantitative polymerase chain reaction (RT-qPCR)

Total RNA from hAESCs, hepatic precursor cells (HPCs), HLCs, and hPH were extracted using Trizol regent. Quantitative real-time PCR was performed with SYBR Premix Ex Taq (TaKaRa) on the ABI StepOnePlus real-time PCR system (Applied Biosystems). All quantitative PCR data were obtained with at least three repeats. GAPDH primers were used as an internal standard. Primer sequences are provided in Table 2.

**Table 2:** Primer sequences for real-time quantitative PCR to detect hepatocyte-specific gene transcripts in hAESCs_,_ HLCs, and hPH

Genes	Sequence
GAPDH	F:5′-CCACATCGCTCAGACACCAT-3′
	R:5′-GCGCCCAATACGACCAAAT-3′
CYP3A4	F:5′GCAAGAAGAACAAGGACAACATAGAT-3′
	R:5′-GCAAACCTCATGCCAATGC-3′
CYP1A2	F:5′-CTCCTCCTTCTTGCCCTTCA-3′
	R:5′-TGTAGAAGCCATTCAGCGTTGT-3′
CYP7A1	F:5′-CTCGAGAGGCCAAAGAAGGA-3′
	R:5′-CTGTCCAGGCTGGTCTTGAAC-3′
CYP2B6	F:5′-GAGAGAGTCTACAGGGAGATTGAACA-3′
	R:5′-GCATTTTGGCTCGGTCATG-3′

### ALB and urea production

Human AESCs were cultured in differentiation medium for 0, 7, and 14 days, respectively, and the supernatant (conditioned medium) was collected. The secretion of ALB was quantified with an ALB enzyme-linked immunosorbent assay (ELISA) kit (Cloud Clone, Nanchang, China). For the measurement of urea contents, the media were collected after 24 h of exposure to 1 mmol/l NH_4_Cl (Sigma) and the urea production was analyzed by using QuantiChrom™ Urease Assay Kit (BioAssay Systems, California, USA). A fresh culture medium supplemented with 1 mmol/l NH_4_Cl was used as a control.

### Glycogen storage staining

After formalin-ethanol fixation, undifferentiated hAESCs, HLCs, and hPH were stained with Periodic Acid-Schiff (PAS; Sigma) staining system and then rinsed with distilled water prior to visualization under a light microscope (IX83; Olympus, Japan).

### Indocyanine green uptake assay

For indocyanine green (ICG; Sigma) uptake assay, the undifferentiated hAESCs, HLCs, and hPH were incubated in the medium supplemented with 1 mg/ml ICG for 1 h at 37 °C and then washed with PBS for three times and visualized under a light microscope (IX83; Olympus).

### Preparation of CCl_4_-induced acute liver failure mouse model and HLC transplantation in mice

To induce acute liver failure, NOD-SCID mice were injected with CCl_4_ (10% in olive oil, Sigma) at the dose of 1 ml/kg body weight by intraperitoneal injection, and the control mice were injected an equal volume of olive oil alone (*n* = 8; olive oil group). After CCl_4_ treatment for 4 h, 2 × 10^6^ HLCs in 0.5 ml of PBS were intravenously injected into the mice to induce acute liver failure (*n* = 8; CCl_4_+HLCs group) and the control mice were administrated with an equal amount of PBS without HLCs (*n* = 8; CCl_4_+PBS group). Recipient animals were sacrificed on third day and seventh day after HLCs/PBS transplantation. A schematic diagram for the time course of the animal experiment is shown in Fig. 5a. Blood and liver tissue samples from all three groups (olive oil, CCl_4_+PBS, and CCl_4_+HLCs) were collected and stored at − 80 °C for further experiments.

In order to analyze whether the HLC transplantation improves the survival rate of ALF mice, the dose of CCl_4_ was raised to 3 ml/kg body wt. Mouse survival rate was calculated at different time points.

### Assessment of liver functions

At third day and seventh day after PBS or HLC transplantation, blood samples were collected from each mouse and then centrifuged at 3500 rpm for 30 min. The serum was collected for measurements of the total bilirubin (TBIL), alanine aminotransferase (ALT), aspartate aminotransferase (AST), albumin (ALB), and alkaline phosphatase (ALP) with an automated biochemical analyzer (Abbott Aeroset, Abbott Laboratories, Chicago, IL, USA). All samples were run in triplicate.

### Histopathology

After 3 days or 7 days of HLC infusion, mice were euthanized by pentobarbital, and heart tissues were isolated and fixed in 4% paraformaldehyde, embedded in paraffin. The cross sections were harvested at 5 μm and stained with hematoxylin and eosin (H&E) and GFP.

### Lentiviral transduction and whole body fluorescent imaging

The lentiviral GFP expression vector pHBLV-IRES-ZsGreen-PGK-puro was purchased from Hanbio (Shanghai, China, http://www.hanbio.net). Lentivirus-containing supernatants were filtered through a 0.22-μm cellulose acetate membrane filter (EMD Millipore, Billerica, MA, http://www.emdmillipore.com), and the viral titer was calculated to be 10^8^ transducing units per milliliter. Viral supernatants were then used to infect hAESCs at passage 2–3 at a multiplicity of infection of 60 in the presence of 6 mg/ml polybrene (Sigma, Aldrich) for 24 h. Subsequently, infected cells were selected with puromycin (3 mg/ml; Sigma-Aldrich).

For the purpose of cell tracking, the GFP-expressing hAESCs were differentiated into HLCs and transplanted into acute liver failure mice. Mice were euthanized after 3 days of HLC transplantation, and the liver, heart, spleen, lung, kidney, pancreas, and brain were harvested and visualized with whole-body fluorescent imaging system (LB983; Berthold, Germany).

### Human-specific genome assay in HLCs, normal mice livers, and HLC-treated CCl_4_-infused mice livers

Genomic DNA was extracted from HLCs, normal mice livers, and HLC-treated CCl_4_-infused mice livers using a GenElute™ Mammalian Genomic DNA miniprep Kits (Sigma). Primer sequences for human-specific gene Alu were designed according to Fu et al. [8]. PCR were carried out in a PCR thermal cycler (Thermo Hybaid), and the products were electrophoresed on a 1.0% (*m*/*v*) agarose gel containing 0.5 μg/ml ethidium bromide for nucleic acid visualization under UV light.

### Statistical analysis

The results are presented as average value ± standard deviation (SD). The Student *t* test was used for between two-group analyses. One-way analysis of variance (ANOVA) was used to compare data among three or more groups. Differences with a *P* value of < 0.05 were considered statistically significant.

## Results

### Identification and characterization of hAESCs

**Fig. 1:**
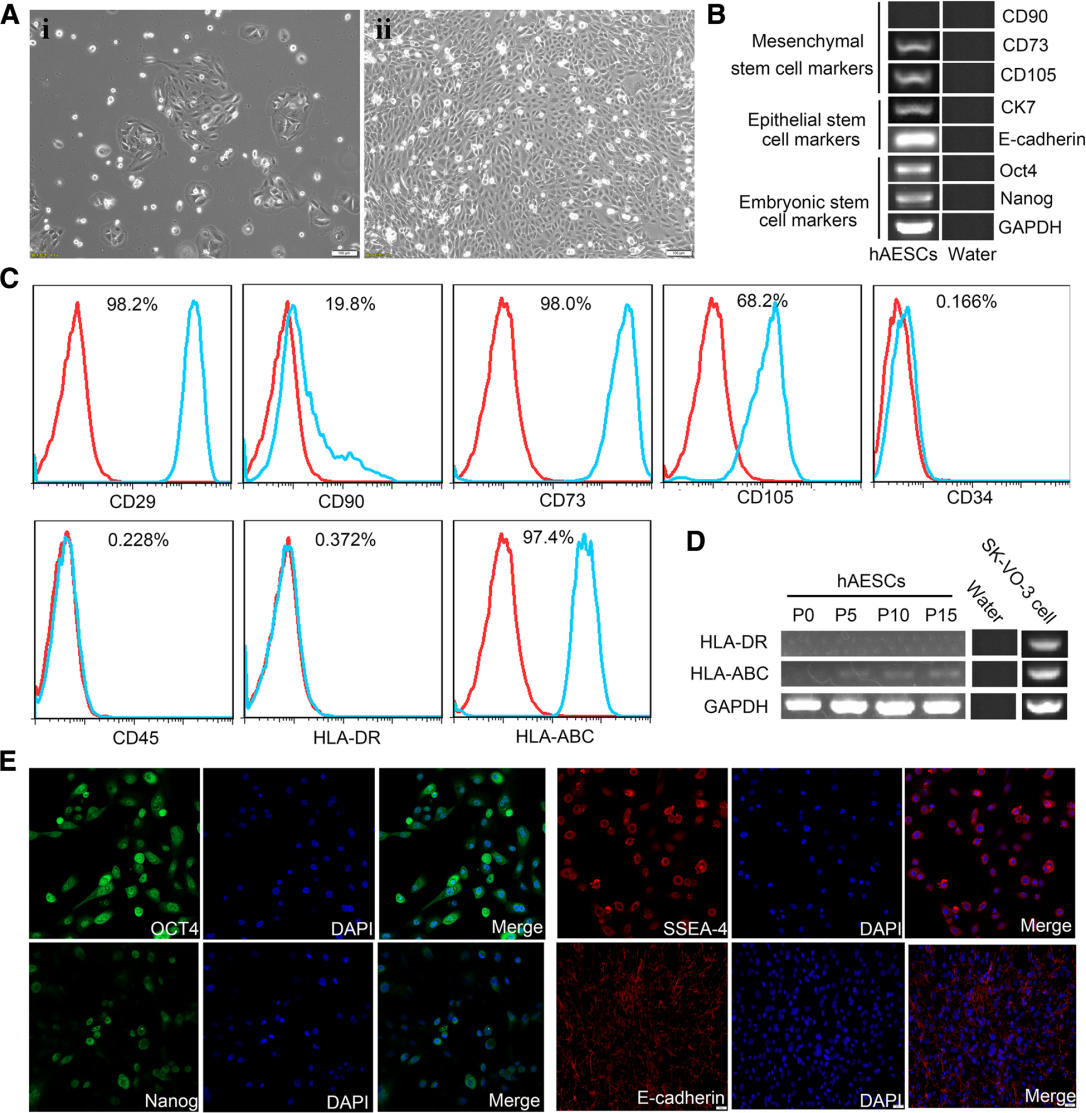
Characteristics of cellular morphology and expressions of markers in hAESCs. **a** Phase-contrast microscopic images of cultured hAESCs without (i) or with EGF (10 ng/ml) (ii). **b** RT-PCR analysis for the expressions of markers in hAESCs. Human AESCs specifically expressed various markers of CD73, CD105, CK7, E-cadherin, Oct4, and Nanog, but not CD90. Water was used as negative control. **c** Detection of surface markers in hAESCs (blue) and in isotype controls (red) by flow cytometry. Human AESCs expressed CD29, CD73, CD105, and HLA-ABC, but not CD90, CD34, CD45, and HLA-DR. **d** RT-PCR analysis for expressions of the major histocompatibility proteins. There were no expression of HLA-DR or low expression of HLA-ABC in hAESCs during passages 0 to 15. Water was used as negative control and ovarian cancer cell SK-VO-3 were used as positive control. **e** Immunofluorescence staining for the markers. All of hAESCs expressed the antigens of Oct4, SSEA-4, Nanog, and E-cadherin

**Fig. 2:**
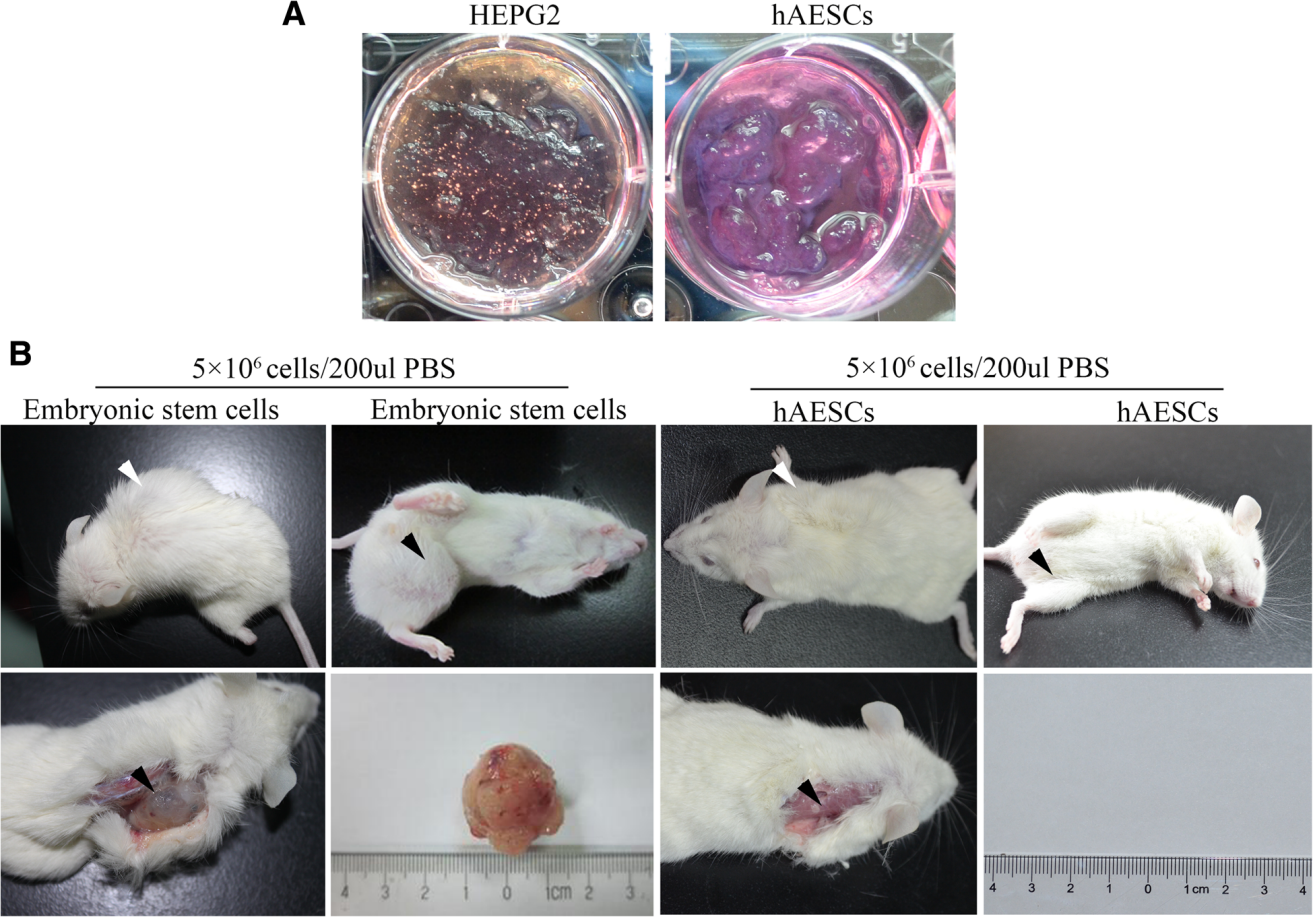
Tumorogenesis of hAESCs in vivo and in vitro. **a** Clonogenicity in soft agar cultures. No colonies were observed within 30 days in the hAESC group. HepG2 cells were used as a positive control. **b** Tumorogenesis in NOD-SCID mice. There was no any tumor formation after 5 months of hAESC injections, in which the cells were injected into the right back and left thigh muscle of NOD-SCID mice. Embryonic stem cells were used as a positive control

Cultured primary and passaged hAESCs showed a cobblestone-like morphology and homogenous growth in monolayers. In the presence of EGF (10 ng/ml), the hAESCs were robustly proliferated and the average doubling time was 2 days (Fig. 1a). The stem cell markers on hAESCs were analyzed by RT-PCR and flow cytometry. As shown in Fig. 1b, c, hAESCs expressed the embryonic stem cell markers Nanog and Oct4, epithelial stem cell markers CK7 and E-cadherin, mesenchymal stem cell markers CD105 and CD73, and major histocompatibility protein HLA-ABC, but not expressed CD90, hematopoietic stem cell markers CD34 and CD45, and major histocompatibility protein HLA-DR. In addition, RT-PCR analysis further confirmed that the hAESCs were negative for HLA-DR and low expression of HLA-ABC (Fig. 1d), indicating that these cells possess low immunogenicity. The expressions of embryonic stem cell surface markers Nanog, Oct4, and SSEA-4 and epithelial stem cell marker E-cadherin on hAESCs were also analyzed by immunofluorescence. Our results showed that hAESCs express all of these pluripotent markers (Fig. 1e), indicating hAESCs have the capability of self-renew as well as multi-lineage differentiation potentials. Soft agar allows for the three-dimensional growth of cell colonies that more closely resembles a tumor than a monolayer. To investigate the tumorigenicity of hAESCs in vitro, Hepg2 (positive control) and hAESCs were seeded into soft agar. In Hepg2 group, many colonies were visible after 30 days; however, no colony was observed in the hAESC group at the same time (Fig. 2a). We next assessed the tumorigenicity of the hAESCs in vivo, in which hAESCs and embryonic stem cells (positive control) were injected into the right back and left thigh muscle of NOD-SCID mice, respectively. We observed no tumor formation in any of the hAESC-injected animals (*n* = 5) over a time period of 20 weeks. In contrast, the large tumors were formed in all mice implanted with embryonic stem cells within 8 weeks (Fig. 2b).

### Induction of hAESCs into hepatocyte-like cells in vitro

**Fig. 3:**
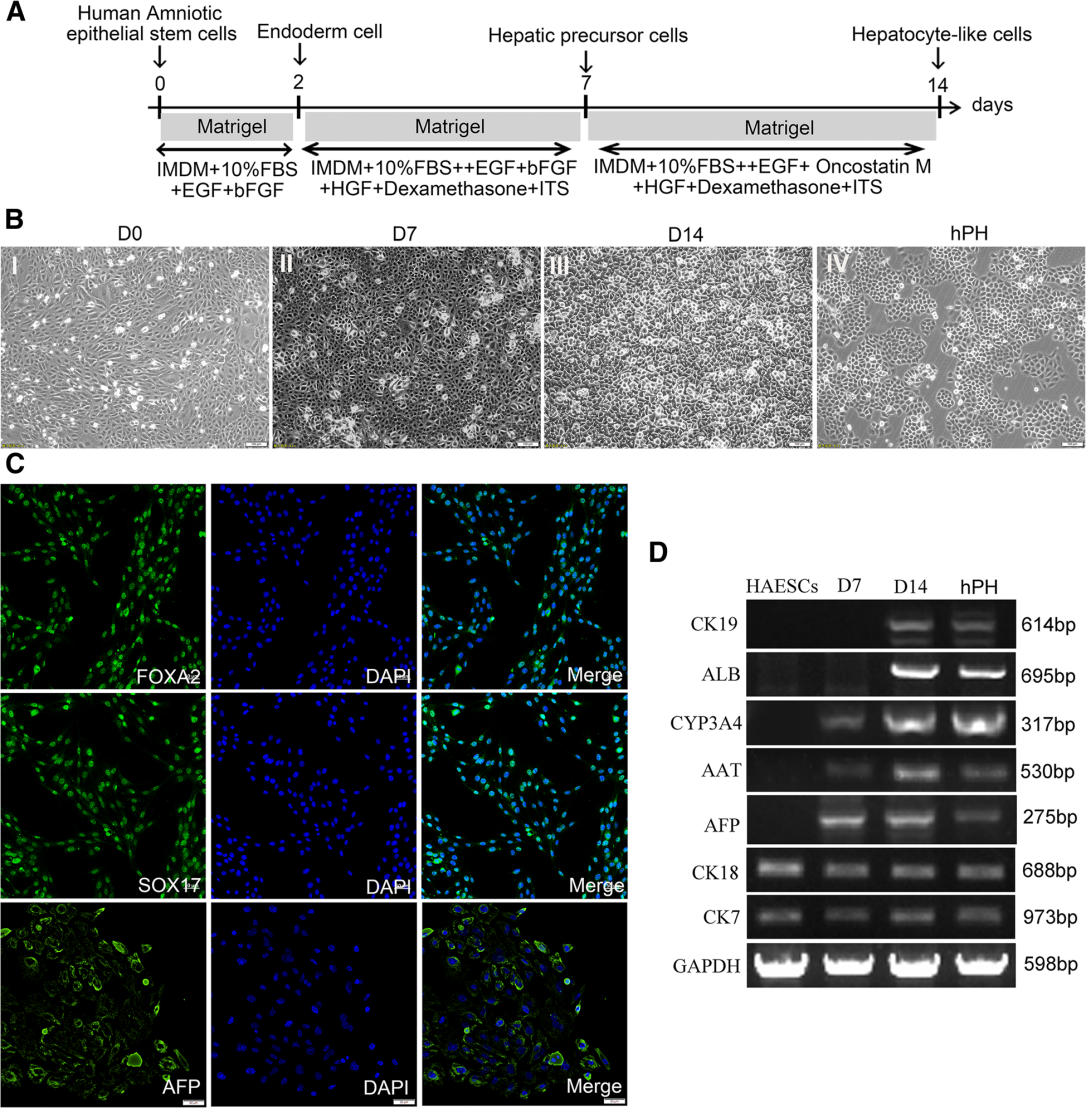
Differentiation of hAESCs into hepatocyte-like cells. **a** Schematic diagram of hAESCs differentiation into hepatocyte-like cells. **b** Images of the sequential morphological changes from hAESCs to hepatocyte-like cells. (I) Morphology at the preinduction stage of hAESCs. (II) The cell morphology has become polygonal in shape after 7-day hepatic lineage commitment medium induction. (III) At day 14, the cell morphology had become cuboidal in shape. (IV) The morphology of hPH. **c** Immunocytochemical examination of endoderm cells and hepatic precursor cells. The vast majority of induced cells expressed the definitive endoderm marker FOXA2 and SOX17 at day 2 and AFP at day 7 after induction of the differentiation procedure. **d** Expressions of the hepatocyte markers in the various stages of differentiation. After 0, 7, and 14 days of induction, the expressions of hepatocyte markers CK7, CK18, AFP, AAT, CYP3A4, ALB, and CK19 of hAESCs differentiated cells and hPH were analyzed by RT-PCR

hAESCs were induced to differentiate into hepatocyte-like cells by a three-step, 14-day protocol as described in Fig. 3a. In this protocol, hAESCs were allowed to reach approximately 90% confluence on Matrigel-coated dishes (Fig. 3b (I)), followed by treatment with endodermal induction medium in the presence of EGF and bFGF on day 0 for 2 days. Immunofluorescent staining showed that most of the cells were positive for the endoderm marker Sox17 and FOXA2 (Fig. 3c), indicating that the hAESCs were differentiated into endoderm cells. Following the endodermal induction step, the cells were treated with the hepatogenic induction medium containing EGF, bFGF, HGF, dexamethasone, and ITS for 5 days. The cell morphology was changed to a polygonal shape (Fig. 3b (II)) from cobblestone-like shape (Fig. 3B (I)). RT-PCR analysis revealed that the cells highly expressed the early liver cell marker AFP and had low expression of mature hepatocyte markers CYP3A4 (a major metabolizing enzyme) and alpha-1-antitrypsin (AAT) (Fig. 3d). Immunofluorescent staining also revealed that the vast majority of these cells were positive for AFP (Fig. 3c). These results indicated that the hAESCs were able to differentiate into hepatic precursor cells (HPCs). Finally, the medium was changed to maturation medium containing EGF, Oncostain M, HGF, dexamethasone, and ITS for 7 days, which resulted in cell morphology changing into the cuboidal shape (Fig. 3b (III)) similar to primary human hepatocytes (Fig. 3b (IV)). We define these cells as hepatocyte-like cells (HLCs). RT-PCR analysis showed that CK7 and CK18 were expressed in hAESCs and were stably expressed during the process of induction. CYP3A4 and AAT were expressed in the early stage of differentiation and were elevated along with increased incubation time. On day 14, the mature hepatocyte markers, ALB and CK19, were detected in hepatocyte-like cells (Fig. 3d). These results indicated that we had successfully obtained hAESC-derived hepatocytes using a non-transgenic protocol within 14 days.

### Functional characterization of the hAESC-derived HLCs

**Fig. 4:**
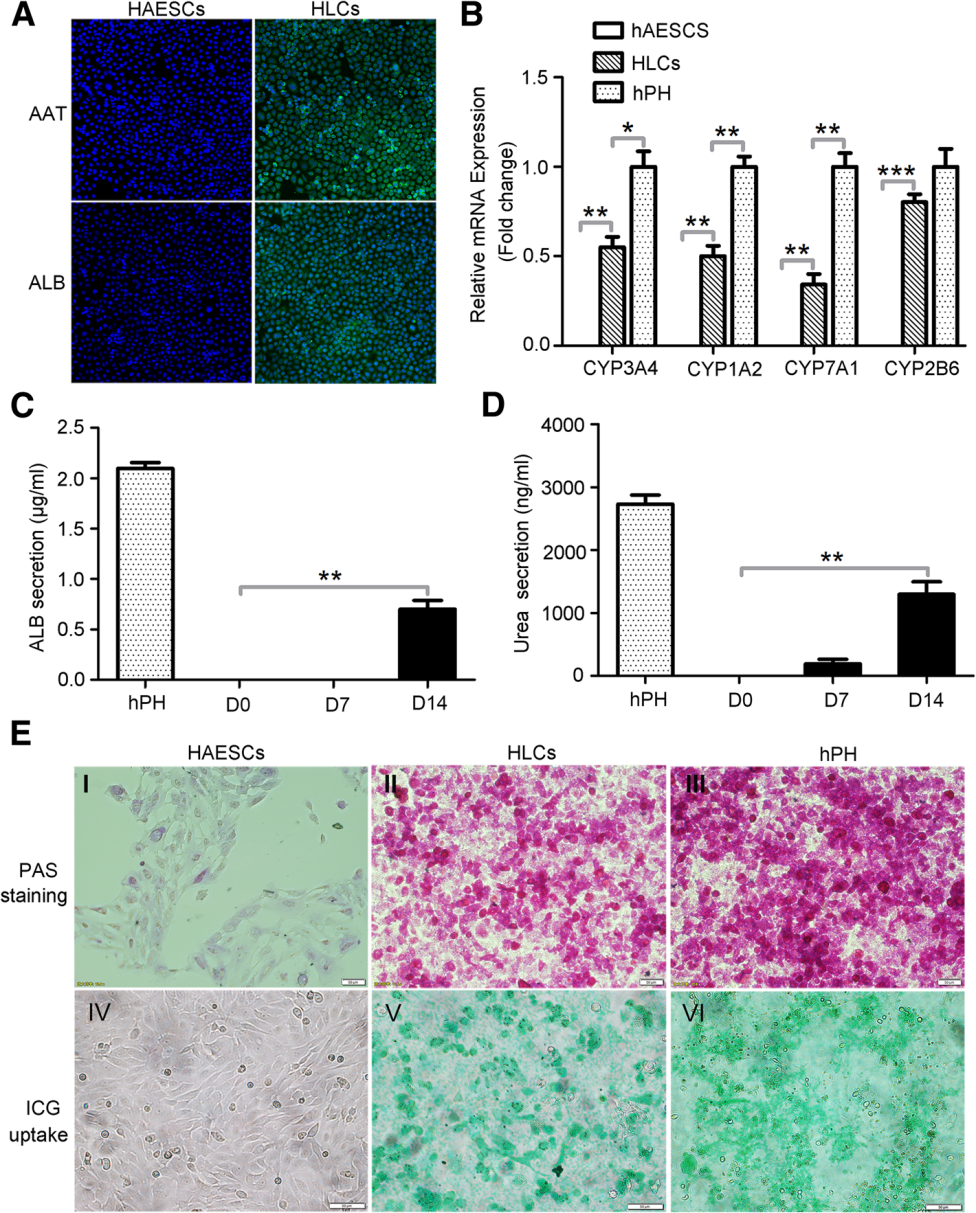
Functional analysis of HLCs derived from hAESCs. **a** Immunofluorescence analysis of AAT and ALB in hAESCs and HLCs. There were no expressions of AAT and ALB in hAESCs, and the HLCs specifically expressed both AAT and ALB after induction of 14 days. **b** Expressions of hepatocyte-specific markers. The mRNA expressions of CYP3A4, CYP1A2, CYP7A1, and CYP2B6 were detected by RT-qPCR in hAESCs, HLCs, and hPH, and the results showed that HLCs specifically expressed all of these markers. **c** Secretion of albumin. The secretion of albumin was determined by ELISA in the culture medium of hPH, hAESCs, and HLCs, and the results showed that the HLCs specifically secreted albumin after induction of 14 days. The hPH cells were used as the positive control. **d** Biochemical analysis of urea. The urea production was also secreted in culture medium of HLCs after induction of 7 and 14 days. The hPH cells were used as the positive control. **e** PAS staining and ICG uptake. The glycogen storage and ICG uptake function of hAESCs, HLCs, and hPH were analyzed by PAS staining (I, II, III) and ICG uptake (IV, V, VI), respectively, and the results showed that HLCs can be specifically stained by PAS and uptaken ICG. Significance was measured via a two-way ANOVA. *P < 0.05, *P < 0.01, ***P < 0.001

We measured the protein expressions of AAT and ALB, the CYP enzyme activity, the secretion of ALB, the urea production, glycogen synthesis, and the ICG uptake to test whether the HLCs assumed the functional attributes of mature hepatocytes. The ALB and AAT synthesis assays are the specific tests for the presence and metabolic activities of mature hepatocytes. Immunofluorescent staining showed that most of HLCs (more than 90%) were both positive for mature hepatocyte markers AAT and ALB. In contrast, hAESCs were negative for AAT and ALB (Fig. 4a). Cytochrome P450 (CYP450) enzymes are the most important enzymes involved in the metabolism of drug in the liver. Their activities are used to evaluate drug metabolism of hepatocytes. We performed RT-qPCR analysis to examine the activities of CYP enzymes CYP3A4, CYP1A2, CYP7A1, and CYP2B6 in hAESCs, HLCs, and hPH. Our results demonstrated that the HLCs exhibited CYP3A4, CYP1A2, CYP7A1, and CYP2B6 activities similar to that found in hPH and that the expression level of the enzymes was remarkably higher than hAESCs (Fig. 4b). As shown in Fig. 4c, the measurement of ALB secretion in the supernatants was only noted on day 14 of differentiation with a value of 0.72 ± 0.13 μg/ml. Urea assay showed that the HLCs could remove ammonia from culture medium. The urea concentration gradually increased along with the differentiation period from a value of 193.33 ± 122.20 ng/ml on day 7 of differentiation to a value of 1296.67 ± 345.01 ng/ml on day 14 of differentiation. The ALB secretion and urea production were not observed in hAESCs. The hPH were used as a positive control (Fig. 4d). To further characterize the glycogen storage function of HLCs, the presence of stored glycogen was determined by PAS staining. Glycogen was stained with magenta and was seen in HLCs and hPH, but not in hAESCs (Fig. 4e, I–III). In addition, HLCs also exhibited high levels of ICG uptake, similar to that of hPH, whereas no ICG uptake was detected in undifferentiated hAESCs (Fig. 4e, IV–VI). Our results clearly indicate that the HLCs derived from hAESCs were functionally similar to matured primary hepatocytes.

### HLC transplantation protected hepatic injuries from CCl_4_-induced acute liver failure in mice

**Fig. 5:**
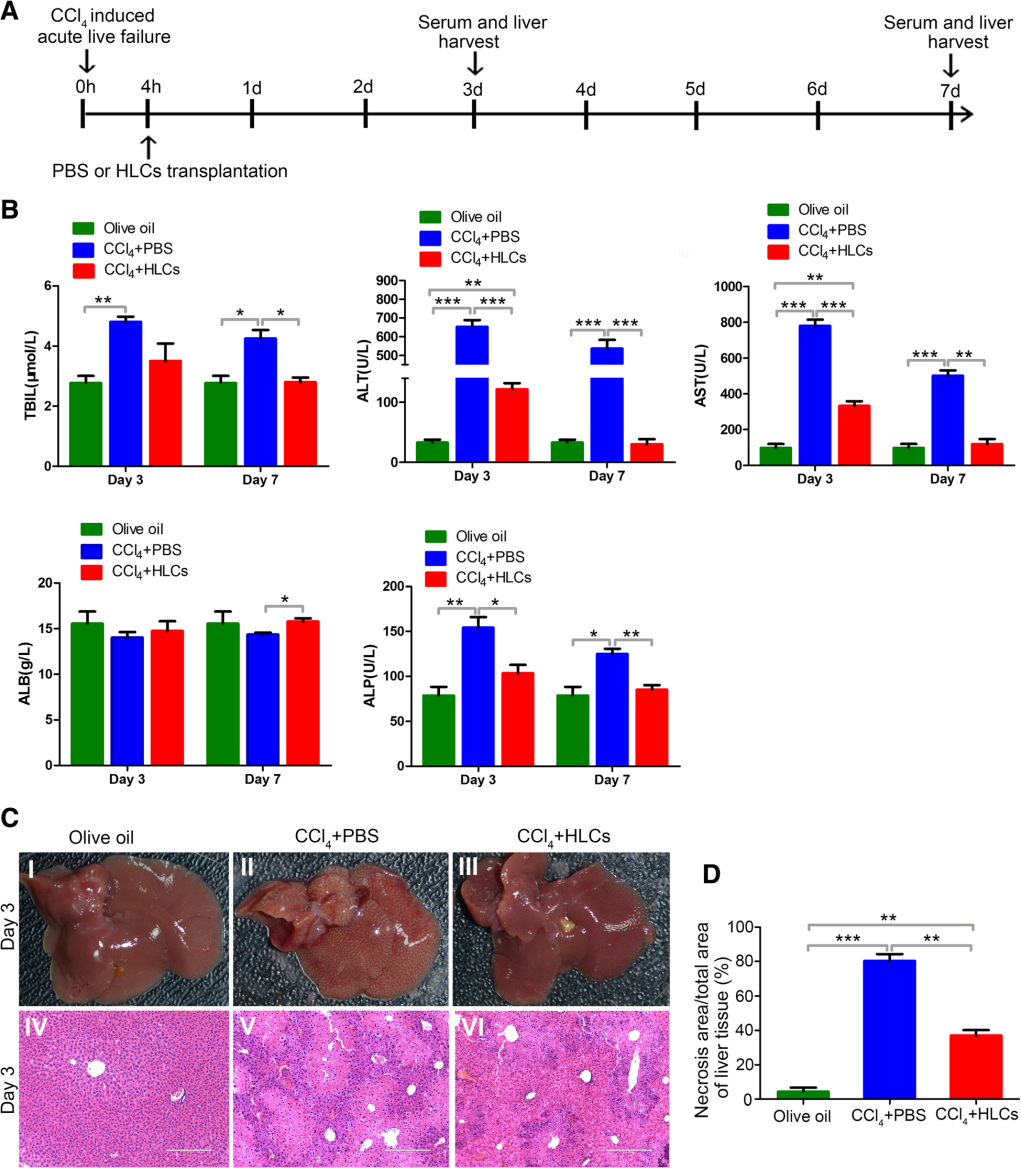
hAESC-derived hepatocyte-like cells improve the liver functions and reduce hepatic damage in ALF mice. **a** Experimental schematic of CCl_4_-induced acute liver failure in the NOD-SCID mouse model. The experiments were conducted in three groups including the olive oil group, CCl_4_+PBS group, and CCl_4_+HLCs group. **b** Examinations of liver function. Some liver functional parameters including TBIL, ALT, AST, ALB, and ALP were determined in olive oil, CCl_4_+PBS, and CCl_4_+HLC group mice after 3 days and 7 days of HLC transplantation. **c** H&E staining of liver tissue. HLC injection led to notable improvements in the liver tissue structure at the day 3. **d** Measurements of necrosis area. The necrosis area of five random, nonoverlapping fields was quantitatively measured in liver tissues of CCl_4_-induced ALF mice from different experimental groups. Significance was measured via a two-way ANOVA. *P < 0.05, *P < 0.01, ***P < 0.001

To evaluate the therapeutic potential of HLCs in ALF mice, the functional HLCs were transplanted into mice with ALF. The transplantation protocol is illustrated in Fig. 5a. NOD-SCID mice were given an intraperitoneal injection of CCl_4_, and olive oil-treated mice were used as a control. 2 × 10^6^ HLCs in 500 μl of PBS or PBS was intravenously administrated into the mice after 4 h of CCl_4_ injection. After 3 days of HLCs transplantation, the live functions were evaluated by measurements of serum TBIL, ALT, AST, ALB, and ALP. Compared with those in the olive oil control group, the values of TBIL, ALT, AST, and ALP were significantly higher in CCl_4_+PBS-treated mice. There were increases of 1.73-fold for TBIL, 19.98-fold for ALT, 8.01-fold for AST, and 1.96-fold for ALP in CCl_4_-injury mice. However, these biomarkers showed comparatively lower increases (1.27-fold for TBIL, 3.72-fold for ALT, 3.42-fold for AST, and 1.31-fold for ALP) on day 3 in the CCl_4_+HLCs group. Compared with the CCl_4_+PBS group, HLC injection reduced the levels of TBIL, ALT, AST, and ALP (*P* < 0.05) and increased the level of ALB. At day 7, serum ALB levels were significantly higher in the mice treated with HLCs than that in the PBS-treated mice (*P* < 0.05), whereas TBIL, ALT, AST, and ALP levels were significantly lower in the CCl_4_+HLCs group mice than CCl_4_+PBS group mice. The mice receiving HLC transplantation showed nearly normal levels of these biomarkers of liver function. There were no significant differences of the biomarkers between CCl_4_+HLCs group and olive oil group (Fig. 5b). After 3 days of CCl_4_ injection, as compared with the olive oil-treated mice (Fig. 5c (I)), the CCl_4_-treated mice displayed submassive hepatocyte necrosis, extensive vacuolar degeneration, and inflammatory cell infiltration in most of the zones of the parenchyma (Fig. 5c (II)). Remarkably, transplantation of HLCs dramatically attenuated the liver damage (Fig. 5c (III)). Histopathologic analysis of liver sections also confirmed the therapeutic potential of HLCs in reducing the CCl_4_-induced hepatic necrosis (Fig. 5c (IV-VI), d). Taken together, our results showed that HLC transplantation via the caudal vein significantly improved liver function and protected hepatic injuries from CCl_4_-induced liver damage in mice.

### hAESC-derived hepatocyte-like cells can engraft in live parenchymal and rescue lethal fulminant hepatic failure

**Fig. 6:**
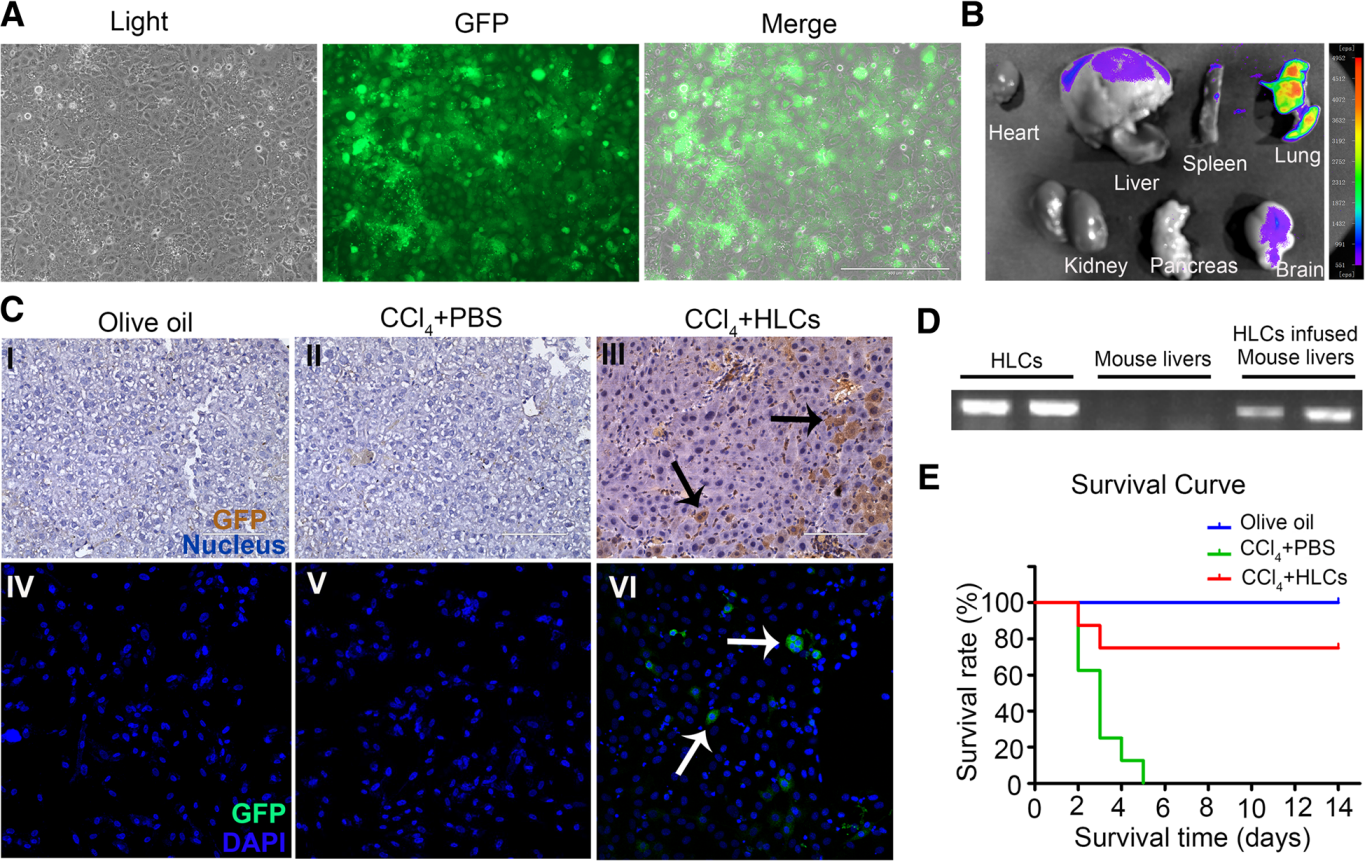
The distribution and therapeutic effects of HLCs in lethal acute liver failure mice. **a** Representative images of GFP-expressing HLCs. **b** The distribution of GFP marked HLCs in vivo of acute liver failure mice on day 3 after transplantation. HLCs mainly distributed in the lung and liver. **c** Immunohistochemical staining and immunostaining analysis of GFP-expressing HLCs in liver tissue. The results showed that GFP-positive cells were detected in injured liver after 7 days of infusion. **d** Human-specific Alu gene were analyzed by RT-PCR using genomic DNA extracted from HLCs, normal mice livers, and HLC-infused CCL_4_-infused mice livers. **e** Survival curves of CCl_4_-induced ALF in mice. The CCl_4_-induced ALF NOD-SCID mice were administrated intravenously with 2 × 10^6^ HLCs (CCl_4_+HLCs group) or PBS (CCl_4_+PBS group), and the death rates were determined within 14 days. The olive oil-treated mice (olive oil group) were used as a normal control (*n* = 8 in each group)

For the purpose of cell tracking, the GFP-labeled hAESCs were prepared for inducing differentiation of HLCs in vitro. 2 × 10^6^ GFP-expressing HLCs (Fig. 6a) in 500 μl of PBS or PBS was intravenously transplanted into mice after 4 h of CCl_4_ injection. To verify whether HLCs migrated to the sites of liver injury, the heart, liver, spleen, lung, kidney, pancreas, and brain were harvested at 3 days after cell transplantation and visualized with whole-body fluorescent imaging system. As shown in Fig. 6b, HLCs mainly located in the lungs and liver, indicating part of HLCs migrated into injured liver. To further investigate whether the transplanted cells were engrafted in liver parenchyma of the recipients, the GFP was used to detect human HLCs in mouse liver. Recipient mice were sacrificed on day 7 after transplantation. The immunohistochemical staining (Fig. 6c (I–III)) and immunostaining analysis (Fig. 6c (IV–VI)) showed the presence of human GFP-positive cells in the liver parenchyma of recipient animals, indicating that the hAESC-derived hepatocyte had been engrafted in recipient liver parenchyma. A genomic DNA assay demonstrated that Alu, a human-specific gene, was detected in both HLCs and the CCL_4_-treated mice liver infused with HLCs, but not in normal mice liver. The result further confirmed the presence of HLCs in recipient mice liver (Fig 6d). To assess the therapeutic potential of hAESC-derived HLCs, a model of lethal fulminant hepatic failure caused by CCl_4_ in NOD-SCID mice was prepared. The dose of CCl_4_ was raised from 1 ml/kg body weight to 3 ml/kg. In the CCl_4_+PBS group, all animals died within 5 days after administration of CCl_4_ (0 of 8 mice survived), whereas in the CCl_4_+HLCs group, 75% (6 of 8 mice survived) of the animals were rescued from the transplantation of 2 × 10^6^ HLCs (Fig. 6e). Taken together, our data suggest that treatment with HLCs significantly improved the survival rate of mice that received a lethal dose of CCl_4_ compared with PBS-treated animals.

## Discussion

The amnion, which is composed of cuboidal and columnar epithelial cells and fibroblast-like mesenchymal cells, has been proposed as an important source of stem cells. The placental tissue is a medical waste after birth, and large quantities of hAESCs can be easily obtained from the donor’s amnion and cultured in vitro without ethical and legal issues. In addition, hAESCs have broader differentiation potential and can differentiate into all three germ layers: ectoderm (neural cells) [23, 30], mesoderm (cardiomyocyte) [31], and endoderm (liver [23, 32, 33] and pancreas [23, 34–36]) in vitro. Furthermore, hAESCs have low immunogenicity and no tumorigenicity [37]. All of these characteristics in hAMESCs make them as a promising source for regenerative medicine.

In the present study, we observed that three core pluripotent proteins (OCT4, SSEA-4, and Nanog) were stably expressed in hAESCs from passage 1 to 5, indicating that the pluripotent characteristics can be maintained during the period of the tested time in hAESCs. Recently, studies have shown that E-cadherin had significant effect on regulating pluripotent and self-renewal signaling pathways in stem cells [38]. Our results showed that the hAESC populations were almost 100% positive for E-cadherin, indicating that these cells were not contaminated with other cellular types from the amnion or chorion. Our data also revealed that the hAESCs expressed high levels of mesenchymal stem cell markers CD29, CD73, and CD105, and the morphology of hAESCs was gradually changed to fibroblast-like shape from cobblestone-like shape (P0-P5) when the passage was increased, indicating that the hAESCs might be transformed into mesenchymal stem cell under the culture condition in vitro. The human leukocyte antigen (HLA) system represents the loci of genes that play a crucial role in determining donor-recipient immune compatibility in organ transplantation [39]. Ours and other’s results showed that there was a very low or no expression of HLA class I (HLA-A, HLA-B, HLA-C) and II (HLA-DR and DQ) in hAESCs [40], suggesting that there is the weak immunogenicity and potential immune tolerance after transplantation of hAECs. In addition, we also observed that hAESCs have no tumorigenicity both in vivo and in vitro, suggesting that the cells may be more safe for clinical applications.

Recently, several investigators have described the differentiation of hAESCs into cells that display hepatic characteristics. Yang et al. observed that human amniotic epithelial cells can be differentiated into hepatocyte-like cell, which expressed hepatocyte-specific proteins ALB and CYP3A4 [33]. Fabio et al. found that HLCs derived from hAESCs expressed hepatocyte markers at levels comparable to those of fetal hepatocytes [32]. Although these results are promising, but no research groups have researched whether these HLCs possess the functional properties of mature hepatocytes in vivo and in vitro [32]. In our study, the HLCs derived from hAESCs not only expressed AAT, ALB, AFP, CYP3A4, and CK19, but also have the ability to produce urea, secrete ALB, uptake ICG, store glycogen, and express CYP enzymes activity, indicating these cells were functionally competent in vitro. The hepatocytes in the liver are maintained in a three-dimensional environment and supported by surrounding cells. When cultured in vitro in two-dimensional environment, hPH also quickly lose activities [41]. So, it is no surprise that the HLCs in our study showed much lower levels of CYP enzymes activity, ALB secretion, and urea generation when comparing with hPH.

In 2008, Tom et al. found that mesenchymal stem cell (MSC)-derived hepatocytes, transplanted by either intrasplenic or intravenous route, engrafted recipient liver and rescued liver failure. To determine whether the route of administration plays an important role in the therapeutic regimen, they compared the effectiveness of intravenous with that of intrasplenic transplantation. They demonstrated that intravenous transplantation of hepatocytes was more effective in rescuing liver failure than intrasplenic transplantation [42]. Banas et al. [43] and Fu et al. [8] also found that after transplantation into NPG mice with acute liver failure through the tail vein, human adipose-derived stem cell-derived hepatocytes could improve the liver function and prolong the life. In addition, Zhou et al. demonstrated that intravenous transplantation of hepatocyte-like cells from umbilical cord-derived MSCs into nude mice with CCl_4_-induced fulminant liver failure and acute liver injury not only improved serum parameters and the alternations of liver histology, but also improved survival rate of mice in severe hepatic failure [44]. There are also many other papers demonstrated that delivery of hepatocyte-like cells through intravenous injection was safe and effective [45–47]. To our knowledge, no attempts have so far been made to determine whether the hAESC-derived HLCs have the ability to improve liver function and rescue the acute hepatic failure mice. In this study, we transplanted hAESC-derived HLCs into CCl_4_-induced liver failure mouse through the tail vein and found that the liver function was significantly improved in the HLC transplantation group than in the control group. More importantly, the survival rates were also significantly higher in the HLC transplantation group than in the PBS group (75% vs. 0%), indicating that the hAESC-derived HLCs have great clinical value in the treatment of acute liver injury. Imaging detection, immunohistochemistry, and immunofluorescence have shown that GFP-expressing HLCs have the ability to engraft into CCl_4_-treated livers, where they may act locally in the injured area to reduce hepatocyte necrosis, extensive vacuolar degeneration, and inflammatory infiltration. These results suggest that injection of hAESC-derived HLCs into acute liver failure NOD-SCID mouse through intravenous injection was safe and effective.

The mechanisms by which HLCs improve live function and survival rate of ALF mice have not been defined. POLL et al. found that the MSCs enhance tissue regeneration either by engraftment and differentiation of the donor cells at the site of injury or by paracrine mechanisms [48]. Zagoura et al. found that the secreted molecules from amniotic fluid mesenchymal stem cells (AF-MSCs) or hepatic progenitor-like cells derived from AF-MSCs have a significant benefit in the treatment of acute hepatic failure [15]. So, we think the following possible mechanisms may account for the observed effects of HLC transplantation. (1) The transplanted HLCs may fuse with hepatocytes and replacement of the damaged hepatocyte. (2) Transplanted HLCs may support and activate liver stem cells or progenitor cells, which then further initiate endogenous cell proliferation and differentiation. (3) Transplanted HLCs may secrete multiple cytokines and growth factors that will inhibit inflammation and accelerate liver restoration. Future studies should be conducted for elucidating the detailed mechanisms.

## Conclusion

In this study, we established a new protocol for generating ample functional hepatocyte-like cells from hAESCs within 14 days. The safety and effectiveness of hAESC-derived HLCs were also demonstrated for the first time in mouse model of ALF. Our study provides strong evidence that transplantation of the hAESC-derived HLCs could be a potential therapeutic strategy for treatment of ALF in clinical applications.

## Abbreviations

AAT: Alpha-1 antitrypsin
AFP: Alpha-fetoprotein
ALB: Albumin
ALF: Acute liver failure
ALP: Alkaline phosphatase
ALT: Alanine aminotransferase
AST: Aspartate aminotransferase
CCl_4_: Carbon tetrachloride
CD: Cluster of differentiation
CK: Cytokeratin
CYP: Cytochrome P450
ELISA: Enzyme-linked immunosorbent assay
ES cell: Embryonic stem cell
FoxA2: Forkhead box a2
GAPDH: Glyceraldehyde 3-phosphate dehydrogenase
hAESCs: Human amniotic epithelial stem cells
HBSS: Hanks’ balanced salt solution
HLA-ABC: Human leukocyte antigen class I
HLA-DR: Human leukocyte antigen-antigen D related
HLCs: Hepatocyte-like cells
HPCs: Hepatic precursor cells
hPH: Human primary hepatocytes
hPSCs: Human pluripotent stem cells
ICG: Indocyanine green
MSCs: Mesenchymal stem cells
Oct-4: Octamer-binding transcription factor 4
OLT: Orthotopic liver transplantation
PAS: Periodic acid-Schiff
RT-PCR: Reverse transcription-polymerase chain reaction
SOX17: Sex-determining region Y box 17
SSEA-4: Stage-specific embryonic antigen 4
TBIL: Total bilirubin
